# Evolution, opportunity and challenges of transboundary water and energy problems in Central Asia

**DOI:** 10.1186/s40064-016-3616-0

**Published:** 2016-11-04

**Authors:** Lidan Guo, Haiwei Zhou, Ziqiang Xia, Feng Huang

**Affiliations:** 1 International River Research Centre, Hohai University, Nanjing, 211100 China; 2 Business School, Hohai University, #8 West Focheng Road, Jiangning District, Nanjing, 211100 China; 3 Jiangsu Provincial Collaborative Innovation Center of World Water Valley and Water Ecological Civilization, Nanjing, 211100 China; 4 College of Hydrology and Water Resources, Hohai University, Nanjing, 210098 China

**Keywords:** Central Asia, Transboundary water conflict, Energy contradiction, Water-energy cooperation, Silk Road Economic Belt

## Abstract

Central Asia is one of the regions that suffer the most prominent transboundary water and energy problems in the world. Effective transboundary water-energy resource management and cooperation are closely related with socioeconomic development and stability in the entire Central Asia. Similar to Central Asia, Northwest China has an arid climate and is experiencing a water shortage. It is now facing imbalanced supply—demand relations of water and energy resources. These issues in Northwest China and Central Asia pose severe challenges in the implementation of the Silk Road Economic Belt strategy. Based on the analysis of water and energy distribution characteristics in Central Asia as well as demand characteristics of different countries, the complexity of local transboundary water problems was explored by reviewing corresponding historical problems of involved countries, correlated energy issues, and the evolution of inter-country water-energy cooperation. With references to experiences and lessons of five countries, contradictions, opportunities, challenges and strategies for transboundary water-energy cooperation between China and Central Asia were discussed under the promotion of the Silk Road Economic Belt construction based on current cooperation conditions.

## Background

Transboundary water resources may become the root of tension and fierce competition among countries because of the increasing global shortage of fresh water resources. Central Asia is one of the regions that are suffering prominent transboundary water and energy problems in the world. The transboundary water resource problem is closely related with the socioeconomic development and stability of the whole Central Asia. The interaction between water and energy issues in Central Asia is a critical factor that makes the transboundary water problem complicated. Five countries in Central Asia are beset with numerous contradictions in distribution and demand of water and energy resources. Similar with Central Asia, Northwest China is characterized by an arid climate, water shortage, and uneven spatial–temporal distribution of water resources. It faces an imbalanced supply–demand of water and energy resources. Many scholars have studied transboundary water problems in Central Asia (Deng and Long 2011; Gao 2012; Klotzli 1997; Li and Liu 2005; Mosello 2008; Xia et al. 2013; Yang and Wang 2010; Yao and Zhou 2014) as well as related climate changes (Bernauer and Siegfried 2012; Li and Liu 2005; Renat 2007; Malsy et al. 2012), energy problems (Song 2014; Zhang 2008), energy cooperation between China and Central Asian countries and Russia (Xu 2013; Zhang 2009a, b). Existing studies and research on the transboundary water problem in Central Asia have focused on water allocation, water management, water conflict and cooperation, and climate changes. Studies about energy problem in Central Asia have emphasized on energy distribution, policy, cooperation (Mao 2013), and diplomacy (Zhu 2011). Nevertheless, only a few research studies on contradiction and coordination between water and energy resources in Central Asia have been reported. The contradiction and coordination between water and energy resources are among the key internal causes of the complexity of the transboundary water problem. With the accelerated promotion of the proposed “Silk Road Economic Belt” strategy, water and energy problems in Central Asia and Northwest China will pose severe challenges against further socioeconomic development of involved regions. In this paper, the complexity of the transboundary water problem in Central Asia is discussed based on water and energy distribution characteristics as well as demand features of different countries. Later, disputes and cooperation practices of Central Asian countries in transboundary water and energy resources are analyzed, and corresponding experiences and lessons have been summarized. Research results provide references for China to achieve water and energy security in the construction of the Silk Road Economic Belt through effective cooperation with Central Asia states.

## Transboundary water-energy distribution characteristics in Central Asia

### Distribution and demand characteristics of water resources

The geographic scope of Central Asia includes the Kazakhstan hills, Turan lowland, Tianshan Mountains, and one part of the Pamirs. It mainly covers Kazakhstan, Kyrghyzstan, Tajikistan, Turkmenistan, and Uzbekistan as well as Afghanistan in the West Asia and some territory of the Aral Sea in Iran. Main bodies of water in Central Asia include inland lakes (e.g., Caspian Sea, Aral Sea, Balkhash Lake, Issyk-Kul Lake, and Alakol Lake), inland rivers (e.g. Amu Darya, Syr Darya, Ili River, Kara Tal River, Emin River, Talas River, Chu River, and Ural River), and one external river (Irtysh River). All of these rivers or lakes are inland and are mainly replenished by melted snow from high mountains and summer rainfall. Moreover, these lakes and rivers basically belong to transboundary river basins between Central Asian countries or their neighbor countries.

Available water resources in Central Asia are mainly surface water, underground water, and return water. Surface water resources are rivers and, which are supplemented by melted snow from high mountains and summer rainfall. Influenced by the geographic location, fresh water resources in Central Asia are distributed extremely and unevenly (Weinthal 2006). Mountains in Kyrgyzstan, Tajikistan, and the northeast Kazakhstan are connected with plateaus. Rich snow and glacial water resources have developed various rivers and lakes, forming a dense river network. It is the runoff formation region in Central Asia and possesses relatively higher total water resources. On the contrary, Turkmenistan, Uzbekistan, and the central south of Kazakhstan are plains, lowlands and deserts, which have a sparse river network and few water resources (Hu et al. 2014). The most prominent transboundary water problem in Central Asian is the fight for water resources of the Aral Sea among five Central Asian countries. According to hydrologic data of the Aral Sea basin from 1911 to 2000, the annual average runoff in the Aral Sea basin was 112.609 billion m^3^, including 77.093 billion m^3^ of the Amu Darya and 34.076 billion m^3^ of the Syr Darya. Table 1 shows the uneven distribution of surface water resources of the Aral Sea basin in five Central Asian countries. Tajikistan and Kyrgyzstan in the upstream possess over 2/3 (43.4 and 25.1%) of surface water resources of the whole Central Asia, while Uzbekistan, Kazakhstan and Turkmenistan in the downstream share the rest or about 1/3 of surface water resources.

**Table 1:** Surface water resources composition of the Aral Sea in the basin countries

Country	Syr Darya river		Amu Darya river		Aral sea Bain	
	Annual mean runoff (billion m^3^/year)	Ratio (%)	Annual mean runoff (billion m^3^/year)	Ratio (%)	Annual mean runoff (billion m^3^/year)	Ratio (%)
Kazakhstan	2.426	6.5			2.426	2.1
Kyrgyzstan	27.605	74.2	1.604	2.0	29.209	25.1
Tajikistan	1.005	2.7	49.578	62.5	50.583	43.4
Turkmenistan			1.549	1.9	1.549	1.2
Uzbekistan	6.167	16.6	5.056	6.4	11.223	9.6
Afghanistan and Iran			21.593	27.2	21.593	18.6
Total	37.203	100	79.28	100	116.583	100

Almost all Central Asian countries are typical agricultural countries. Agriculture occupies large proportions of their national economic structures. In arid and semi-arid regions in Central Asia, precipitation is inadequate to maintain local rain-fed agriculture and irrigation agriculture becomes the only one choice. Agricultural water is the main water usage in Central Asia. Water-consuming industrial crops (e.g., cotton and rice) accounted for 90% of agricultural water usage. Water usage of farm land per capita amounts to nine times of that in developed industrial countries (Li 2009). In the centralized planned economic system of the Soviet Union, a complementation between the water resources advantages of upstream Kyrgyzstan and Tajikistan and the energy resources (including petroleum, natural gas, and coal) advantages of downstream Uzbekistan, Kazakhstan, and Russia is formed. Upstream reservoir water resources are mainly used for farmland irrigation in the downstream. However, after the collapse of the Soviet Union, energy structure, conflicts of agricultural mode, and economic structure between upstream and downstream countries emerged. The fight for water resources among five Central Asian countries were exposed gradually. Kyrgyzstan and Tajikistan would hold upstream river water for hydroelectric generation as their pillar industry, which further intensified the water shortage of the downstream countries. This irrational industrial structure and the economic development mode in the region caused structural contradictions of water demands among different countries.

### Distribution and demand characteristics of energy resources

Central Asia is rich in strategic energy resources, including petroleum, gold, natural gas, various minerals, and strategic materials (e.g., uranium). It is the third biggest petroleum reserve region in the world, and is second only to the Persian Gulf and Russia Siberia (Zhang 2009a, b). Although there are abundant energy resources in Central Asia, these resources present an extremely uneven spatial distribution (Weinthal 2006). Reserves, outputs, and consumptions of petroleum and natural gas in Central Asian countries are shown in Tables 2 and 3 (Bernauer and Siegfried 2012). In Central Asia, Kazakhstan, Turkmenistan, and Uzbekistan possess rich energy resources, including petroleum, natural gas, and coal.

**Table 2:** Reserves, outputs and consumptions of petroleum in five Central Asian countries in 2012

Country	Verified reserves (million ton)	Proportion of the world’s verified reserves (%)	Outputs (million ton)	Proportion of the world’s outputs (%)	Consumptions (million ton)	Proportion of the world’s consumptions (%)
Kazakhstan	3900	1.8	81.3	2.0	12.8	0.3
Turkmenistan	100	Less than 0.05	11.0	0.3	4.8	0.1
Uzbekistan	100	Less than 0.05	3.2	0.1	3.9	0.1
Kyrgyzstan	–	–	–	–	–	–
Tajikistan	–	–	–	–	–	–

The data source is BP Statistical Review of World Energy 2013; notation “–” denotes no reliable data

**Table 3:** Reserves, outputs and consumptions of natural gas in five Central Asian countries in 2012

Country	Verified reserves (billion m^3^)	Proportion of the world’s verified reserves (%)	Outputs (billion m^3^)	Proportion of the world’s outputs (%)	Consumptions (billion m^3^)	Proportion of the world’s consumptions (%)
Kazakhstan	130	0.7	19.7	0.6	9.5	0.3
Turkmenistan	1750	9.3	64.4	1.9	23.3	0.7
Uzbekistan	110	0.6	56.9	1.7	47.9	1.4
Kyrgyzstan	–	–	–	–	–	–
Tajikistan	–	–	–	–	–	–

The data source is BP Statistical Review of World Energy 2013; notation “–” denotes no reliable data

Of these countries, the Kazakhstan economy is dominated by petroleum, natural gas, coal, and husbandry. As the biggest petroleum country in Central Asia, Kazakhstan has the highest proven petroleum reserves and annual output than any other Central Asian country. Natural gas is the major energy resource in Turkmenistan which is located in the southwest of Central Asia and the east coast of the Caspian Sea. Its land petroleum resources and oil and gas resources in the Caspian Sea occupy a strategic energy position in Central Asia. Particularly, oil and gas resources in the Caspian Sea have attracted worldwide attention. Uzbekistan also possesses tremendous oil and gas resources. Its 63% territorial area is in the oil and gas concentration belt along the Caspian Sea. Uzbekistan is one of the top ten countries in global natural gas exploitation, and its annual natural gas output is only next to that of Turkmenistan in Central Asia.

Tajikistan and Kyrgyzstan are high-mountain countries. Although they possess a great deal of water resources and waterpower resources, they lack oil and gas resources and face great difficulties in exploration and exploitation. Therefore, they substantially rely on energy imports. Without the economic value of petroleum and natural gas reserves, Tajikistan would greatly depend on Uzbekistan and Turkmenistan. For Kyrgyzstan, the domestic fossil fuel resource reserves are far from enough to satisfy the needs of the Kyrgyz people. Approximately 95% of annual demand for crude oil, natural gas, coal, and petroleum products depends on imports (Zhang 2010). These fossil fuel resources are imported from Kazakhstan and Uzbekistan, and are principally used for electricity and heat generation in thermal power plants during the dry season (winter). Electricity is an important industry in Kyrgyzstan, but is also one of the important export commodities. It mainly exports electricity to members of the Commonwealth of Independent States, especially neighboring countries, such as Kazakhstan, Tajikistan, Uzbekistan, and Russia. At present, extensive hydropower development is not only an important premise of natural energy security but also an important component of the national economic development strategy of Kyrgyzstan. However, the hydropower generation structure is significantly restricted by uneven time distribution and great inter-annual variation of water resources. The annual power generation is unstable, high during the wet year (or wet season), but low in the dry year (or dry season), thus resulting in the unstable exportable electricity.

## Historical evolution of transboundary water-energy contradictions in Central Asia

### Water-energy resources management during the former Soviet Union

#### Water-energy transformation was implemented under the coordination of the central government of the former Soviet Union, aiming to maximize benefits of all parts

Mountain topography in the upstream of Amu Darya and Syr Darya is a good place for water storage and hydropower generation. It is the main water production region in the basin, but it lacks land resources and energy resources. Downstream regions are flat and are main water-consuming regions, but there are rich land resources and energy resources. Such water and energy distribution characteristics do not match water and energy demands by local economic development. Giving comprehensive considerations to the national and regional overall development plans and economic layout, the former Soviet Union planned water conservancy facilities in the upstream of the Amu Darya and the Syr Darya, but emphasized the role of irrigation agriculture and industry in downstream regions and providing energy resources, and industrial and agricultural products to upstream regions. This implementation realized the goal of a balanced development of the whole region (Abdolvand et al. 2015). The former Soviet Union government offered financial support for the construction and maintenance of upstream water conservancy facilities. Consequently, upstream Tajikistan controlled 8% water volume of the Amu Darya and 9% water volume of the Syr Darya, whereas Kyrgyzstan controlled 58% water volume of the Syr Darya. Water conservancy facilities in Tajikistan are mainly for water storage for the purpose of large-scale farmland irrigation in the downstream Uzbekistan, Turkmenistan, and Kazakhstan. Farmland irrigation in downstream countries highly depends on water reservoirs in the upstream Kyrgyzstan and Tajikistan. Although the upstream Kyrgyzstan and Tajikistan have an energy shortage, they can import petroleum, natural gas, and coal for heating from Uzbekistan, Kazakhstan, and Russia.

#### Division of labor was determined and centralized management of water resources was implemented

During the early stage of the former Soviet Union, the principle of “division of labor” was set up in Central Asia, striving to develop Central Asia into a single agricultural production base. For example, Uzbekistan and Kazakhstan are major cotton production bases, whereas Kyrgyzstan is an agricultural production base and the major power generation base. Although such division of labor caused water allocation conflicts among union republics of Central Asia, these conflicts could be effectively coordinated by the centralized control of the former Soviet Union. During the former Soviet Union, the central government adopted centralized management of water resources in Central Asia and practiced the water quota and loss compensation system. This realized the vision of a reasonable allocation and usage of water resources among Central Asian countries, and relieved imbalanced supply–demand relations of water and energy resources among union republics of Central Asia.

### Complicated water-energy interweaving contradictions of Central Asian countries after the collapse of the Soviet Union

The water resources management policy of Central Asia during the former Soviet Union has caused a profound impact on Central Asia after the collapse of the Soviet Union. After five Central Asian countries became independent, the fiercest water fight in Central Asia was between upstream and downstream countries of the Aral Sea. Countries began to pay close attention to the allocation of water resources of the Amu Darya and the Syr Darya and corresponding tributaries. Meanwhile, the intertwined energy and water problems complicated the transnational issues in Central Asia.

#### The water resource allocation problem was not solved effectively due to the absence of an agreed instruction center

After the collapse of the Soviet Union, although the previous water conservancy facilities and water allocation system were retained, Central Asian countries have gained sovereignty and have their respective national interests. Resources (including water recourses) became their own properties. Currently, the combination of Soviet central planning and fractious independent states has created numerous challenges (Mosello 2008). In the early stage of the independence of the five Central Asian countries, they all focused efforts to developing their own national economy and controlled their national resources strictly. They once made significant efforts to establish effective organizations and signed a series of agreements to coordinate and manage water resources in Central Asia. However, they emphasized on independent foreign policies and tried to protect their own interests during the execution of agreements, causing various contradictions. Furthermore, some countries were in chaos, which hindered the mutual communication and understanding of many problems. They acted arbitrarily in water resource decisions rather than cooperated with each other, failing to solve the transboundary water allocation problem in Central Asia effectively.

#### Water-energy conflicts between upstream and downstream countries intensified continuously

Due to the uneven water distribution in Central Asia and different interests of the various countries, water conflicts among upstream, midstream, and downstream countries of the Aral Sea basin took place continuously. The upstream Kyrgyzstan and Tajikistan possess rich water resources and hydropower resources, but they have few available land resources and energy resources. They fall behind others in terms of economic development and are identified as poor countries in Central Asia. The downstream Uzbekistan, Kazakhstan, and Turkmenistan, though faced with a severe water shortage, covers extensive land area and are rich in oil, gas, and energy resources. They are considered countries in Central Asia. The cotton-oriented economic development of Soviet planners made these downstream countries consume most of the water resources of the region, and accordingly had produced environmental depletion and degradation (Klotzli 1997). Restricted by the water resource management system of the former Soviet Union, upstream countries could not generate adequate electricity during winter, which could not be offset through hydroelectric generation. However, they could obtain the necessary energy supply from the downstream countries under a central unified management system. After the collapse of the Soviet Union, many irrigation projects and hydroelectric generation projects that were built during the former Soviet Union turned into transnational projects. Previous water conservancy facilities in the upstream became properties of upstream countries, but served the entire region. Without adequate farmland, upstream countries could not produce self-sufficient grains nor afford such heavy expenditures under a sluggish national economy. Kazakhstan and Uzbekistan, which received several benefits from these rivers in the downstream, were neither willing to undertake such expenditures nor provide any compensation to the upstream countries. Since upstream countries are short of energy resources and other natural resources, they treat water as a strategic commodity. They changed the running patterns of reservoirs in the upstream of the Amu Darya and Syr Darya, and continued to develop hydropower resources in the territory to meet their own electricity demands (Renat 2007). Compensatory energy resources to upstream countries decreased significantly, which were inadequate to maintain the normal operation of thermal power plants and ensure daily electricity utilization and winter heating for residents. As a result, upstream countries have recognized water as a strategic commodity, and have resorted to storing water continuously during spring or the snow-melting period during summer and make hydroelectric generation in winter to meet normal electricity demand. This situation resulted in water storage of upstream reservoirs in winter and the sharp reduction of irrigation water to downstream countries in the summer. Hence, the farmland area and crop output in downstream countries were reduced accordingly, accompanied with declining living conditions of residents and ecological deterioration. Downstream countries have restricted water consumption and water resources development of upstream countries through various treaties and agreements, but upstream countries continued to enhance hydropower development, ignoring these treaties and agreements. These developments cause evident conflicts of water and energy demands between the upstream and downstream countries.

Since fossil fuel resources in Central Asia are distributed unevenly between upstream and downstream countries, the exchange between water resources and energy resources was free of charge during the Soviet Union period. However, some of the latter agreements did not consider energy problems and mutual dependence. Involved parties often broke promises when they are in advantageous positions. Therefore, a water distribution agreement which is independent from the energy distribution problem could not be developed continuously. Upstream countries suffer from a lack of energy supply from the downstream countries, while downstream countries are short of water supply from upstream countries. None is willing to make concessions, resulting in the continuous intensifying water-energy conflicts between upstream and downstream countries in Central Asia.

## Current cooperation situations of Central Asian countries in transboundary water resources and future development

### Current cooperation situations

With the regional socioeconomic development, Central Asian countries in the Aral Sea basin implemented different degrees of cooperation under continuous water conflicts. Five independent Central Asian countries have experienced from conflict and fighting to coordination and cooperation with respect to allocation, utilization, and protection of water resources (Sulanman and Silati 2014). The transboundary water management mechanism in Central Asia evolved from a regional organization to government control, and then to seeking international assistance. Central Asian countries had established various regional organizations to coordinate and solve water resources problems, such as the Transboundary Water Conservancy Coordination Committee (1992), International Foundation of the Aral Sea (1993), and International Committee of Aral Sea Issues (1993). They also established the highest-summit meeting mechanism and regular and irregular minister negotiation mechanism to solve the water resources crisis and avoid water conflicts. To address the water and ecological problems in the Aral Sea basin, five Central Asian countries attempted to internationalize the Aral Sea issues, expecting to win more international assistance. In recent years, Central Asian countries claimed to settle and mediate water problems and water conflicts within the Shanghai Cooperation Organization Framework. Since the ecological problem of the Aral Sea occurred, many countries and international organizations have studied the Aral Sea issue and offered Central Asian countries great support. However, water resources problems in Central Asia are caused by complicated historical background and profound actual factors, which are extremely difficult to solve quickly.

### Future development of transboundary water-energy problems

The long-term transboundary water conflicts and imbalanced energy supply–demand in Central Asia have brought various consequences, such as ecological deterioration, land desertification, and the reduction of crop yields, and living quality and health level of the people. If these problems could not be reasonably solved in the next two or three decades, they may not only intensify conflicts of Central Asian countries but may also have serious socio-political consequences. As an example, the Aral Sea has undergone rapid desiccation and salinization since 1960 as a consequence of unsustainable expansion of irrigation (Micklin 2016), which had severe negative impact on both the hydrology and ecology status of the water body and the society development in the basin. Although 25 years have passed since the breakup of the Soviet Union, issues of transboundary water and energy resources in Central Asia have not been solved, and water cooperation is still in its formative years. What would be the state after another 25 years or longer? Considering the close correlation between water and energy problems in Central Asia and the complicated interest relations among different countries, Central Asian countries should disregard previous enmity, enhance mutual understanding and cooperation, coordinate interest relations of involved parties, target the maximization of common interest, perfect economic structure, and establish a scientific and reasonable water allocation system and water-energy exchange mechanism. These actions should be pursued to maintain peace, stability, and the development of the region. Only in this way could they solve the transboundary water allocation and utilization problems as well as correlate energy supply–demand issues effectively, use and protect limited water resources reasonably, and achieve mutual development.

## Opportunities and challenges of water-energy cooperation between China and Central Asian countries

### Opportunities

#### The transboundary water cooperation between China and neighboring Central Asian countries should be enhanced continuously

The main transboundary rivers between Northwest China and Central Asian countries are Ili River, Irtysh River, Emin River, and Aksu River. Similar to the neighboring Kazakhstan and Kyrgyzstan, Northwest China belongs to an arid inland area of Central Asia and faces prominent conflicts between socioeconomic development and water supply–demand. Although China possesses relative geographical advantages in water resources development, transboundary rivers in Northwest China have been developed and utilized slightly in the past (Hao 2012). Therefore, no evident transboundary water problem between Northwest China and the neighboring countries has taken place. Recently, China cooperated with Kazakhstan and Kyrgyzstan in transboundary river utilization and protection to differing extents. Under the comprehensive development of strategic partnership between China and Kazakhstan, cooperation in the development of transboundary rivers has achieved important progress through the China-Kazakhstan Joint Commission in the field of use and protection of transboundary rivers (Joint Commission), which was established in 2003. In the past two decades, China and Kazakhstan contributed by collaborating on technical issues, negotiating the creation of and process for effective comprehensive mechanism in the use and protection of transboundary water resources. Under the joint efforts of China and Kazakhstan, ecosystems in the Ili River and the Irtysh River are healthy. These rivers are the transboundary rivers that have been protected most effectively in Central Asia. During 2003–2015, 12 meetings of the Joint Commission were held, following the results of which important agreements and outcomes were reached. Work has been launched on the draft Agreement on water apportioning in transboundary rivers between China and Kazakhstan, which is being developed by a special working group established in 2014.^1^ China and Kazakhstan have signed a series of agreements or minutes on joint use and protection of transboundary rivers at the level of the President, Minister, or expert.^2^ The completion of the China-Kazakhstan Khorgos River Fellowship Diversion Pivotal Project (2013) is an important sign of comprehensive China-Kazakhstan transboundary river cooperation. China-Kazakhstan cooperation has become the model of transboundary river cooperation in Central Asia. As to China and Kyrgyzstan, with the aim of further enhancing the scale and level of economic cooperation, both sides are accelerating the negotiations on the inter-governmental cooperation agreement of transboundary river (Aksu River) utilization and protection. All of these friendly transboundary river cooperation practices have laid the foundation for further expanding water-energy cooperation in Central Asia.

#### The energy imports of China from Central Asian countries can grow gradually

With the gradual growth of global energy demands, five Central Asian countries have become more and more positive in attracting foreign investments. On one hand, they reinforce exploration to increase reserves and take the initiative to seek cooperation to cope with periodic energy shortages and to reduce dependence on non-renewable energy resources. On the other hand, they expand infrastructure construction for energy transmission more actively and realize diversified energy exports continuously, thus taking the initiative on energy supply and to expand new international markets. Therefore, they can achieve a stable balance between energy exports and foreign currency earnings (Zhang 2012).

Energy security is a key global problem. The energy demand of China, which is experiencing rapid economic development, is increasing day by day. China takes various forms of international cooperation as one of the important means to solve the energy security problem. The energy cooperation between China and Central Asian countries plays an extremely important role (Zhang 2009a, b).

China makes full use of its close geographical position to reinforce and expand energy cooperation with Central Asian countries. The energy imports of China from Central Asian countries have grown gradually in recent years. Central Asian countries have become one of the important energy strategic partners of China. China mainly exports light industrial products (e.g., textile) to Central Asian countries, and, in turn, imports crude oil, natural gas, and industrial raw materials from them. Such complementary resources and trade structure facilitate economic and trade contact between China and Central Asian countries. The export commodity structure of Central Asian countries have only changed slightly in the past. Energy resources and minerals are still the major export commodities of Central Asian countries. For instance, the principal export products of Kazakhstan are petroleum and natural gas, whereas the main export products of Turkmenistan include natural gas, petroleum products, textile, and electricity. Uzbekistan mainly exports natural gas. According to statistics of the China Customs Statistical Yearbook (2001–2011) (Bernauer and Siegfried 2012), the value of energy trade between China and Central Asian countries grew continuously. Particularly, the value of energy trade between China and Central Asian countries achieved rapid growth in 2007 and 2010 since the opening of the China-Kazakhstan oil pipeline in 2006 and the Central Asia-China natural gas pipeline in 2009.

#### The “Silk Road Economic Belt” strategy may bring huge development space

On September 17th, 2013, Xi Jinping, the Chinese president, stated during his visit to Kazakhstan that China and Kazakhstan shall adopt an innovative cooperation mode and construct the “Silk Road Economic Belt” together, to reinforce economic ties of Asian-European countries and expand the development space. This Economic Belt covers a vital Asian-Pacific region in the east, a Central Asia rich of resources in the middle, and developed European economic entities in the west. Countries along this Economic Belt have strong economic complementation and great potential for mutually beneficial cooperation. With attractive geopolitical interests, the “Silk Road Economic Belt” will expand strategic space for the economic development of China significantly and is conducive to the development of Xinjiang and the West China. It could promote China-Central Asia cooperation in economics, water resources, and energy resources, thus providing strategic support for the steady development of the Chinese economy. Meanwhile, it will facilitate economic and social development of countries along the belt. Under this background, Central Asian countries with rich resources and strategic geographic positions maintain a unique status in the world economy. However, they also desire regional economic cooperation based on the Silk Road Economic Belt to solve the long-term small economic growth (Gang and Ren 2015). For example, they can carry out cooperation in advancing scientific research in technologies such as water-saving irrigation, enhanced education and personnel training, and investment of infrastructure maintenance. Thus, the strategy may provide a broader platform and great opportunities for Eurasian countries along the road.

### Challenges

While the “Silk Road Economic Belt” strategy brings historical opportunities for the socioeconomic development of China and Central Asian countries, further reinforcing trans-regional cooperation, it also brings about challenges, such as water and energy security, to related countries.

#### Dry climate, water shortage, and uneven spatial and temporal distribution of water resources

In arid and semi-arid regions, water resources are key strategic resources that have profound a influence on geopolitical relations among countries in the region. The Asian hinterland is a region short of water resources historically. Northwest China and Central Asia covered in the “Silk Road Economic Belt” have similar arid climate and experience water shortage, which can restrict socioeconomic development greatly (Guo et al. 2015; Shi and He 2015). Given the uneven spatial–temporal distribution of water resources, human activities further intensify the prominent imbalance between water supply and demand and the vulnerable ecosystemt (Chen et al. 2011, 2012). Because of different geographic positions, countries in the Aral Sea basin possess significantly different quantities of water and land resources. With tremendously high mountains, Kyrgyzstan and Tajikistan occupy about 80% of the water volume of the whole basin, but their farmland area is only 13% of the land resources in the basin. Territories of Kazakhstan, Turkmenistan, and Uzbekistan are mostly plains and lowlands. They possess few water resources but extensive farmland area. Agricultural development in Kazakhstan, Turkmenistan, and Uzbekistan depends highly on upstream water. Similarly, Northwest China is also faced with extremely uneven water and land distribution, water shortage, and difficult development and utilization of water resources during socioeconomic development. The increasing pressure from population growth and climate change in Central Asia and Northwest China has substantially decreased the total water resources in the region (Siegfried et al. 2012). This situation, in turn, may bring the region into a precarious situationin the future, where the decreasing water availability coexists with the increasing water demand.

#### Acute structural imbalance of water supply and demand

In Central Asia and Northwest China, precipitation is inadequate to maintain local rain-fed agriculture. Irrigation agriculture becomes the only choice of countries (or regions) in the river basin. Agriculture is the main water user. Water competition between the economic system and ecosystem is very acute, and structural water shortage has become an important constraint against sustainable economic and social development of these countries (or regions). Under the centralized planning economic system of the Soviet Union, upstream countries of the Aral Sea basin (Kyrgyzstan and Tajikistan) and downstream countries (Uzbekistan and Kazakhstan) complement each other. Upstream countries offer water resources to downstream countries for farmland irrigation, while downstream countries supply energy resources (petroleum, natural gas, and coal resources) to upstream countries. After the collapse of the Soviet Union, conflicts of energy structure, agriculture mode, and economic structure between upstream and downstream countries became increasingly prominent. With population growth and the economic rehabilitation of five Central Asian countries, the imbalance of water supply and demand as well as water competition among different countries was further intensified. Similar with most arid regions in the world, Northwest China has problems of an unsustainable water consumption structure and low water-use efficiency and benefit. The economic structure of the “Oasis economy and irrigation agriculture” leads to a high proportion of agricultural water use in Northwest China, valued at 95, 91, 78, and 90% in Xinjiang, Ningxia, Gansu, and the whole Northwest China, respectively (Deng et al. 2011). Moreover, the conflict between water and natural resources utilization and ecological protection in Northwest China sharpened due to the lagging water conservancy construction, which brought severe challenges to water resources management (Li and Kang 2011).

#### Vulnerable ecosystem

In history, large-scale agricultural development and water conservancy construction in Central Asia not only created miracle economic development but also caused big changes in a series of hydrological geographical systems and ecosystems (e.g., river cutoff and lake dry-up). Moreover, irreversible ecological disasters in the Aral Sea Basin (Xia et al. 2013). Arid regions along the Silk Road Economic Belt have vulnerable environment. Ecological security is influenced by local climate change features, water resource distribution characteristics, water development and utilization modes, water-use efficiency, and water resources allocation. Improper water development and utilization will deteriorate the ecosystem. The ecosystem of Northwest China and Central Asia will become one of critical constraints of the establishment of the Silk Road Economic Belt and poses strict requirements on resource development and protection.

#### Complicated transboundary water problem

There are several transboundary rivers in Central Asia. Countries in the basin have been dealing with complicated transnational water conflicts. Particularly, conflicts of upstream hydroelectric generation and downstream irrigation water demand of the Amu Darya and Syr Darya among five independent Central Asian countries have taken place continuously. Northwest China, Kazakhstan, and Kyrgyzstan, which are in the arid inland region of Central Asia, are all faced with the prominent conflict between socioeconomic development and water supply–demand. However, transboundary rivers in Northwest China are far less developed and developed at a later time than local non-international rivers and foreign parts of these transboundary rivers (Hao 2012). In the future, the development and utilization of international rivers in Northwest China will suffer from great pressures from downstream countries and international public opinion. There may be potential transboundary water conflicts between China and neighboring Central Asian countries. Under these circumstances, how China effectively participates in transnational water-energy cooperation with Central Asia is an important strategic problem of China now and in the future.

### Enlightenments

Due to establishment of the Silk Road Economic Belt, involved regions, especially countries in transboundary river basins in the arid region and neighboring countries in the same region, have to cope with water resource problems and environmental problems caused by rapid economic growth. Therefore, countries or regions along the belt urgently need to carry out coordinative development and effective cooperation of water resources. Common economic interests of the countries should align them toward the possibility of cooperation in protecting water resources and ecological security.

As an initiator of the “Silk Road Economic Belt” strategy and a responsible great global power, China has to establish a strategic framework for collaborative development of water resources with Central Asia as soon as possible, based on an existing international water cooperation mechanism and current energy cooperation conditions. Additionally, China should reasonably plan water-energy cooperation projects along the belt, reinforce water and energy cooperation with Central Asian countries, make full use of its water management experiences, technologies and financial strength, learn lessons from water cooperation among Central Asian countries, and build a safe water cooperation corridor oriented to the Silk Road Economic Belt. Implementation of a collaborative water development strategy between China and Central Asian countries is not only an important solution to water resource security along the belt but also the important driving force of promoting socioeconomic growth of countries in Central Asia.

## Conclusion

In Central Asia, large quantities of water are stored in the mountain glaciers of Kyrgyzstan and Tajikistan, while Kazakhstan, Turkmenistan, and Uzbekistan have extensive and mostly unexplored oil and gas deposits. Therefore, Central Asian water management has always been linked to energy issues and security politics. In the face of global change and regional security requirements, there are both opportunities and challenges to solve Central Asian transboundary water-energy issues.

The transboundary water problem in Central Asia is complex, which can be reflected in the following aspects: (a) it has both natural causes and historical reasons; (b) it faces increasing pressures from climate change and population growth; (c) it involves riparian countries that are both sovereign-independent nations with mutual entanglement; (d) conflicts and cooperation coexist in the water-energy nexus. Under these complexities in Central Asia, managing transboundary waters becomes a sensitive and complex task that seems extremely difficult to be solved by the affected players themselves.

As a neighbor of Central Asian countries, China connects with them by mountains and rivers in the northwest. The increasing China-Central Asia cooperation on various aspects (including water) and the continuous promotion of the “Silk Road Economic Belt” strategy may bring promising development to alleviate local transboundary water-energy issues. However, both Northwest China and Central Asian countries are faced with such similar challenges as dry climate, water shortage, imbalance of water supply and demand, and vulnerable ecosystem. Thus, for Central Asian countries, neighboring states, and the international community to devise robust institutions for water cooperation, they should think outside of the ‘water box’ and situate the issue of water within the larger social and economic issues. Central Asian countries can only promote and realize common development by coordinating conflict of interests and reinforcing regional and global cooperation.

## Footnotes

1 Use and protection of transboundary rivers of Kazakhstan and China discussed in Beijing (14 August 2015).
http://www.mfa.kz/index.php/en/consular-issues/press-releases/4531-use-and-protection-of-transboundary-rivers-of-kazakhstan-and-china-discussed-in-beijing.

2 The cooperation between the Republic of Kazakhstan and the Peoples Republic of China in the field of use and protection of transboundary water resources (25 March 2016). http://www.mfa.kz/index.php/en/foreign-policy/current-issues-of-kazakhstan-s-foreign-policy/transboundary-rivers/the-cooperation-between-the-republic-of-kazakhstan-and-the-peoples-republic-of-china-in-the-field-of-use-and-protection-of-transboundary-water-resources.
